# Multivisceral Resection for Locally Advanced Gastric Cancer: A Systematic Review and Evidence Quality Assessment

**DOI:** 10.3390/jcm12237360

**Published:** 2023-11-28

**Authors:** Dimitrios Schizas, Ilias Giannakodimos, Konstantinos S. Mylonas, Emmanouil I. Kapetanakis, Alexandra Papavgeri, Georgios D. Lianos, Dionysios Dellaportas, Aikaterini Mastoraki, Andreas Alexandrou

**Affiliations:** 1 First Department of Surgery, “Laikon” General Hospital, National and Kapodistrian University of Athens, 115 27 Athens, Greece; schizasad@gmail.com (D.S.); iliasgiannakodimos@gmail.com (I.G.); ksmylonas@gmail.com (K.S.M.); apapavgeri@uth.gr (A.P.); dr_kamast@yahoo.gr (A.M.); alexandrouandrea@hotmail.com (A.A.); 2 Department of Thoracic Surgery, “Attikon” University Hospital, National and Kapodistrian University of Athens, 124 62 Athens, Greece; 3 Department of Surgery, University Hospital of Ioannina, 451 10 Ioannina, Greece; glianos@uoi.gr; 4 Third Department of Surgery, “Attikon” University Hospital, National and Kapodistrian University of Athens, 124 62 Athens, Greece; dellapdio@gmail.com

**Keywords:** gastric cancer, gastrectomy, locally advanced, multivisceral resection, systematic review

## Abstract

Patients with locally advanced gastric cancer (LAGC) often require multivisceral resection (MVR) of the involved organs to achieve R0 resection and local disease control. The aim of the present study was to systematically review all available literature on the postoperative and long-term outcomes of MVR for gastric cancer. The PubMed database was systematically searched by two independent investigators for studies concerning MVR for LAGC. In total, 30 original studies with 3362 patients met our inclusion criteria. R0 resection was achieved in 67.77% (95% CI, 65.75–69.73%) of patients. The spleen, colon and pancreas comprised the most frequently resected organs in the context of MVR. Pancreatic fistulae (10.08%, 95% CI, 7.99–12.63%), intraabdominal abscesses (9.92%, 95% CI, 7.85–12.46%) and anastomotic leaks (8.09%, 95% CI, 6.23–10.45%) constituted the most common postoperative complications. Using the available data, we estimated the mean 1-year survival at 62.2%, 3-year survival at 33.05%, and 5-year survival at 30.21% for the entire cohort. The survival rates were mainly correlated with lymphatic invasion, tumor size and patient age. Therefore, gastrectomy, together with MVR, is feasible and may offer a survival advantage compared to gastrectomy alone or no other surgical treatment in a selected group of patients. Consequently, both patient and tumor characteristics should be carefully assessed to optimize candidate selection.

## 1. Introduction

Gastric cancer (GC) represents the fifth-most common malignancy worldwide and remains a major cause of cancer-related mortality [1]. Oncologic resection with negative macroscopic and microscopic margins (R0 resection) along with adequate lymphadenectomy (D2 lymphadenectomy) is considered the gold standard and mainstay of GC treatment [2,3]. Unfortunately, GC typically presents at an advanced stage, and if the tumor invades adjacent structures, extensive surgical resections may be needed to achieve clear resection margins [2,4].

Locally advanced GC (LAGC) accounts for approximately 30–35% of locally advanced gastrointestinal malignancies [5]. Contemporary literature defines locally advanced gastric cancer (LAGC) as any entity larger than early gastric cancer or T2–T4 lesions. Others define LAGC as T3–T4 gastric malignancies requiring neoadjuvant treatment [6,7]. These patients usually have a poor prognosis compared to patients with early-stage GC [8]. Patients with radiologic evidence of T4b disease are potential candidates for multivisceral resection (MVR) [9]. The distal pancreas, spleen, transverse colon and left liver lobe constitute the most frequently resected organs in combination with the gastrectomy specimen during MVR procedures [6]. Unsurprisingly, multivisceral resection carries a high risk of perioperative morbidity and mortality [10,11]. In this context, identifying the patient population that would benefit the most from such radical resections is crucial [12]. Nevertheless, this can be a challenging endeavor, since delineating invasion patterns with the current preoperative staging modalities is not always straightforward. Furthermore, there is a lack of consensus concerning patient characteristics and preoperative factors that can guide the decision-making process with regards to MVR.

The aim of the present study was to systematically review all available literature concerning patient characteristics and short- and long-term survival, as well as postoperative complications, of patients with T4b LAGC undergoing MVR.

## 2. Materials and Methods

### 2.1. Literature Search and Inclusion Criteria

This systematic review was conducted according to the PRISMA (Preferred Reporting Items for Systematic Reviews and Meta-analysis) guidelines and in line with a protocol agreed on by all authors [13]. Two independent investigators (IG and AP) searched the PubMed/Medline and Embase databases for articles reporting on T4b LAGC (last search: 11 October 2021). Boolean operators (AND and OR) in combination with the following keywords were utilized: “gastric cancer”, “gastric neoplasm”, “locally advanced”, “multiorgan resection” and “multivisceral resection”.

Eligible prospective or retrospective studies on MVR for T4b LAGC written in English were retrieved. The references of all the included papers were reviewed to identify additional (potentially eligible) manuscripts using snowball methodology. Any controversies were resolved by a third investigator (KSM). Articles reporting on gastric cancer with distant hematogenous or lymph nodal metastases, studies not written in English, reviews of the literature, case reports, letters to the editor not including original data, in vitro studies, animal studies and abstracts were excluded from this systematic review.

### 2.2. Data Extraction

Data extraction was performed by two independent researchers (IG and AP) using a pre-piloted template. The following variables were collected and tabulated: study characteristics (sample size, type of study and country of origin); patient age; tumor location; tumor size; macroscopic and histological findings; TNM classification; surgical treatment strategies; number of resected or invaded organs; lymphadenectomy and survival rates. Predictive factors were also analyzed.

### 2.3. Statistical Analyses

Continuous variables were summarized as the mean ± SD (standard deviation), while categorical variables were summarized using frequencies and percentages. Relative rates, along with the corresponding 95% confidence intervals (95% CI), were estimated based on the available data for each variable of interest. Statistical analysis was carried out using IBM SPSS Statistics for Windows, Version 24.0, Armonk, NY, USA: IBM Corp.

### 2.4. Assessment of Study Quality

The quality of the included case series was assessed using the tool developed by the National Heart, Lung, and Blood Institute (NHLBI) based on work from the Agency for Healthcare Research and Quality, the Cochrane Collaboration, the United States Preventive Services Task Force, the Scottish Intercollegiate Guidelines Network and the National Health Service Centre for Reviews and Dissemination [14]. The NHLBI scale ranges from 1 to 9, with a score of 1–3 demonstrating poor quality, 4–6 fair quality and 7–9 showing good quality. The mean and SD values for the NHLBI score of this systematic review were calculated. Three independent reviewers (IG, AP and KSM) rated the quality of the included studies, and a synthesis of their reports was performed.

## 3. Results

### 3.1. Article Selection and Patient Demographics

**Figure 1:**
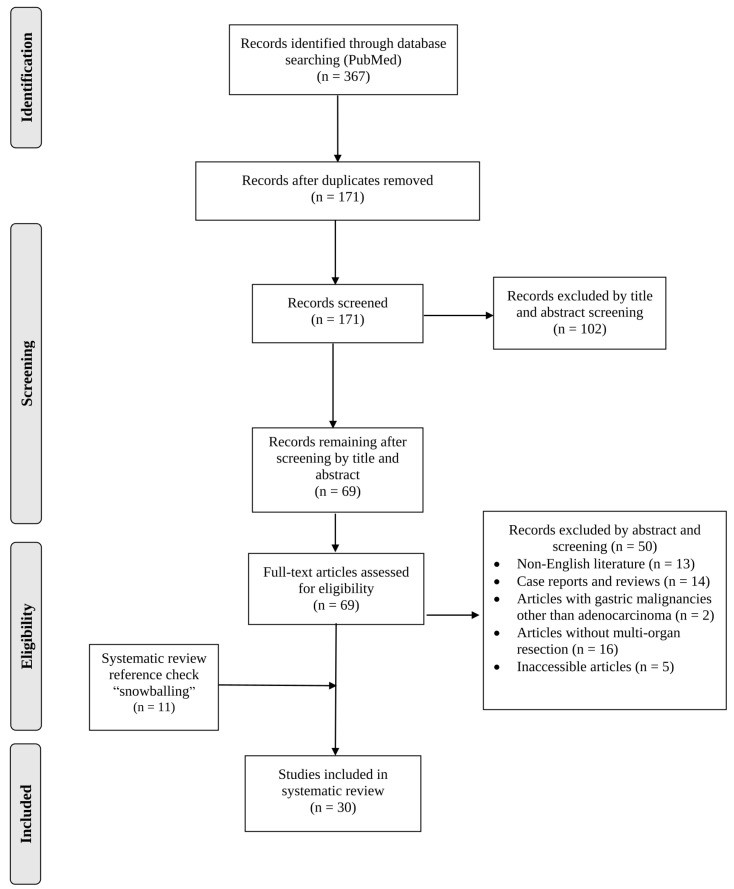
Flowchart of this systematic review.

Our literature search yielded 171 unique articles following the removal of duplicate publications. Overall, 30 articles met the inclusion criteria (Figure 1). The eligible studies were published between 1988 and 2020. In total, 3362 patients were analyzed. Concerning the geographical distribution of the included studies, 56.7% (17 studies) originated from Asian countries, 20% (6 studies) from North America countries, 13.3% (4 studies) from European countries, 3.3% (one study) from an African country, 3.3% (one study) from a South America country and 3.3% (one study) from Australia. Out of the available data, 1612 patients were male and 790 were female, with a 2:1 (male:female) sex ratio. The median patient age ranged from 55.76 to 69.70 years among the studies. However, it should be mentioned that demographic data were not available for 900 patients.

### 3.2. Quality of Evidence Assessment

The mean NHLBI score for the 30 eligible studies that were included in this systematic review was 8.30 (SD: 1.58) (good quality: 28 studies; fair quality: 2 studies). Detailed NHLBI and JBI quality assessments for the included studies are provided in Supplementary Table S1.

### 3.3. Location and Characteristics of Gastric Tumors

The majority of GCs that were treated in the context of MVR were found in the distal part of the stomach (41.3%), while 29.4% were found in the middle part, 23% in the upper stomach and only 6.3% were diffuse. The locations of gastric tumors in patients subjected to multivisceral resection are shown in Table 1. The mean tumor size ranged from 6.84 cm to 9.60 cm. Twenty articles (1200 patients) evaluated the grade of differentiation of the involved GC tumors, with 169 tumors well differentiated (169/1200; 14.08%, 95% CI, 12.23–16.17%), 111 moderate (111/1200; 9.25%, 95% CI, 7.73–11.03%) and 383 (383/1200; 31.92%%, 95% CI, 29.34–34.61%) poorly differentiated. Interestingly, 537 (537/1200; 44.75%, 95% CI, 41.96–47.58%) of the GC tumors were histopathologically classified as undifferentiated.

Out of the 30 included studies, 20 articles described the T stage (1655 patients), and 19 articles reported on the N stage (2094 patients). In total, 221 patients (221/1655; 13.35%, 95% CI, 11.80–15.08%) presented with T3 GC and 1434 patients (1434/1655; 86.65%, 95% CI, 84.92–88.20%) with T4 GC, out of which 615 (615/1434; 42.89%, 95% CI, 40.35–45.46%) had T4a, 625 (625/1434; 43.58%, 95% CI, 41.04–46.16%) had T4b and 194 patients presented with no data concerning T4 stage stratification. Additionally, concerning the nodal status of the included patients, 543 patients (543/2094; 25.9%, 95% CI, 24.1–27.85%) presented without nodal involvement, 413 patients (413/2094; 19.8%, 95% CI, 16.73–19.91%) had N1 involvement, 434 patients (434/2094; 20.7%, 95% CI, 19.04–22.52%) had N2 and 704 patients (704/2094; 33.6%, 95% CI, 31.63–35.67%) had N3 involvement.

### 3.4. Type of Gastrectomy

Total gastrectomy was the most common type of gastric resection that was performed in the context of MVR (1175/2116; 55.53%, 95% CI, 53.4–63%), followed by subtotal gastrectomy, which was performed in 811 (811/2116; 38.33%, 95% CI, 36.28–40.42%) patients. The type of gastrectomy was unspecified in 130 patients. A R0 resection was achieved in 1434 patients that underwent MVR (1434/2116; 67.77%, 95% CI, 65.75–69.73%), while R1 or R2 resections were described in 682 patients (682/2116; 32.23%, 95% CI, 30.27–34.25%). The survival rates of the included patients regarding R resection are presented in Supplementary Table S2.

Out of the available data, gastrectomy along with the resection of one organ was performed on 1500 patients (1500/3362; 44.62%, 95% CI, 42.94–46.30%). Furthermore, gastrectomy combined with the resection of two organs was performed on 585 patients (585/3362; 17.4% 95% CI, 16.16–18.72%), while gastrectomy along with the resection of three adjacent organs was performed on 73 patients (73/3362; 2.17%, 95% CI, 1.73–2.72%). The spleen, colon and pancreas were the most frequently resected organs in the context of MVR. Other excised organs included the small bowels, gallbladder, kidney, adrenal glands and the ovaries. The exact number of resected organs in the reported studies is presented in Table 2.

In total, nine studies, including 985 patients, assessed the role of adjuvant or neoadjuvant therapy in patients that underwent MVR resection. Adjuvant therapy was administered in 798 patients (798/985; 81.02%, 95 CI, 78.44–83.35%), including 642 patients (642/985; 65.18%, 95% CI, 62.15–68.09%) that received adjuvant chemotherapy and 134 patients that received adjuvant radiotherapy (134/985; 13.60%, 95% CI, 11.60–15.89%).

Additionally, 187 patients (187/985; 18.98%, 95% CI, 16.65–21.56%) received neoadjuvant therapy, including 158 patients (158/985; 16.04%, 95% CI, 13.88–18.47%) undergoing neoadjuvant chemotherapy and 12 patients having neoadjuvant radiotherapy (1.22%, 95% CI, 0.67–2.14%), while the type of neoadjuvant therapy received was not mentioned for 17 patients.

### 3.5. Incidence of Postoperative Complications

Out of the 30 analyzed articles, 19 studies reported incidences of various postoperative complications, while only 14 studies described in detail the exact nature of those complications. Of the available data, postoperative complications were reported in 655 patients (33.27%, 95 CI, 31.22–35.38%). The most frequently reported complications were pancreatic fistulae, described in 66 patients (66/655; 10.08%, 95% CI, 7.99–12.63%), intra-abdominal abscesses in 65 patients (65/655; 9.92%, 95% CI, 7.85–12.46%), anastomotic leaks in 53 patients (53/655; 8.09%, 95% CI, 6.23–10.45%) and surgical site infections in 33 patients (33/655; 5.04%, 95% CI, 3.59–7.01%). Postoperative infections were reported in 32 patients (32/655; 4.89%, 95% CI, 3.46–6.84%) and postoperative bleeding in 11 patients (11/655; 1.68%, 95% CI, 0.90–3.02%), while biliary leaks or fistulae were seen in only 4 patients (4/655; 0.61%, 95% CI, 0.18–1.62%).

### 3.6. Survival of Patients Subjected to MVR

Using the available data, we estimated the mean 1-year survival at 65.2% (95% CI, 62.61–67.8), the 3-year survival at 33.05% (95% CI, 30.71–35.5%) and the 5-year survival at 30.21% (95% CI, 28.25–32.25%) for our entire cohort. The mean 1-, 3- and 5- year survival rates of patients undergoing gastrectomy combined with one additional organ were 64.46% (95% CI, 60.39–68.37%), 42.33% (95% CI, 38.19–46.43%) and 32.33% (95% CI, 28.95–35.9%), respectively. As expected, patients who underwent MVR of >1 organ appeared to have inferior survival (1-year survival: 47.1% (95% CI, 40.58–53.63%), 3-year survival: 21% (95% CI, 15.96–26.67%) and 5-year survival: 15.17% (95% CI, 11.3–20%)). The survival rates of patients who were subjected to multiorgan resection are summarized in Table 3.

Out of the available data, N3 gastric tumors and R+ resection were associated with poor survival in 38.44% (95% CI, 34.78–42.23%) and 28.86% (95% CI, 24.82–33.27%) of patients, respectively. The other reported predictors of poor survival included Borrmann type IV in 17.55% (95% CI, 14.18–21.51%), lymphatic or lymphovascular tumors invasion in 44.03% (95% CI: 35.91–52.49%) and pancreatic invasion or resection along with MVR in 38.11% (33.65–42.77%) of the patients. Interestingly, only one study found that adjuvant chemotherapy constitutes an independent indicator of better survival. However, in the multivariate analyses, only a few predictive factors remained as independent indicators of poor survival in the majority of the studies. These included R2 resection, lymph nodal involvement and a positive lymph node ratio. Detailed prognostic factors of the included studies are described in Table 4.

## 4. Discussion

The survival rates of patients undergoing radical procedures for advanced GC remain poor. Undoubtedly, T4b locally advanced gastric lesions that require multiorgan resection are associated with increased morbidity and mortality [12]. That said, the refinement of surgical techniques and optimization of the pre- and postoperative management of GC patients have led to a considerable reduction in postoperative complications and improved patient survival [32]. An increasing body of literature has shown comparable outcomes and survival rates between gastric surgery with MVR and gastrectomy alone [25,27]. In accordance with the published medical literature, this study demonstrated that the prognosis of these patients mainly depends on the number and type of resected organs, size of the primary tumor and lymph nodal involvement. Within this framework, the invasion of gastric tumors into adjacent organs should not constitute a priori a contraindication for potentially curative resections. Our systematic analysis also demonstrated that the 1-year overall survival rates of patients ranged from 53.3% to 88%, 3-year survival ranged from 26.9% to 54.2% and 5-year survival ranged from 17.3% to 40%, suggesting that multiorgan resection in patients with LAGC could be performed safely in experienced hands with acceptable morbidity and mortality rates.

The available data are contradictory with regards to the impact that the type of resected organs exerts on the long-term outcomes of MVR [8]. In this systematic review, the spleen, colon and pancreas constituted the most commonly resected organs during radical surgeries. Colonic resections during MVR for GC are historically associated with a potential survival benefit [32,33]. In a retrospective study of advanced gastric cancer extended to the colon, the 1-, 3- and 5-year overall survival rates were 75.0%, 49.2% and 36.9%, respectively, with a median survival time of 24 months [20]. Building on this knowledge, a recent multicenter trial reported no significant difference between gastrectomy combined with colonic resection alone and gastrectomy along with the resection of other involved organs [8].

For nearly two decades, splenectomy was routinely performed in patients undergoing gastrectomy for malignancies of the fundus, even in the absence of splenic infiltration. According to recent data, this approach offers no major survival benefits and has thus been abandoned. On the other hand, splenectomy in the setting of MVR for T4b lesions has been associated with over 80% and 45% 1-year and 2-year survival rates, respectively [8,22]. Therefore, combining splenectomy with gastrectomy for T4 LAGC appears to significantly improve patient prognoses [8,34,35].

Encouraging outcomes have been reported from series performing pancreatectomies in the context of MVR. In this context, the 1-year survival has been shown to range from 61.5% to 83.8%. The two-year survival has been found to exceed 72%. Although scarce data exist, the 5-year survival rates may approximate 33% [32]. Not surprisingly, pancreatectomy-including MVR increases the incidence of postoperative complications and prolongs the length of the hospital stay substantially [11]. In a study conducted by Piso at al., the long-term oncologic outcomes of patients with combined gastrectomy and pancreatic resection concerned a 5-year survival rate of 19% and a median survival at 13 months [36]. The contradictory findings among published studies may be attributed to the limitations of retrospective studies, including selection bias, the coexistence of potential confounding factors and heterogeneity in practices among different surgeons [31].

Interestingly, a desmoplastic reaction of the surrounding tissues due to widespread GC may be incorrectly recognized as an invasion of the primary tumor to the adjacent organs, leading to an en bloc MVR of the involved structures [11]. This difficulty in recognizing cases with true malignant invasion of the adjacent structures persists due to imaging and staging limitations in delineating tumor anatomy [25]. In this systematic review, 13.27% of the GCs were staged as T3, while the vast majority of patients [86.13%] presented with T4 lesions (42.89% had T4a and 43.58% had T4b). Of note, the majority of included patients that underwent MVR along with gastrectomy had gastric tumors staged as T3 or T4a and were incorrectly considered suitable for MVR. Furthermore, the patient selection for MVR should be more careful, since these patients may present with various postoperative complications. In our systematic analysis, pancreatic fistulae (10.08%), intraabdominal abscesses (9.92%), anastomotic leaks (8.09%) and surgical site infections (5.04%) comprised the most commonly recorded complications. Interestingly, rapid recognition of these complications could be based on various predictive markers, such as the neutrophile/lymphocyte ratio (NLR) for anastomotic leaks in gastrectomies [37].

In large cross-sectional studies, the overall survival of patients with LAGC was estimated at 13.5 months (ranging from 6.2 to 30.3 months), while no significant difference concerning the survival rates between gastrectomy with MVR and gastrectomy alone was reported [6,38]. In our analysis, the estimated mean 1-year survival was 62.2%, 3-year survival was 33.05% and 5-year survival was 30.21%. Interestingly, the impact of the number of resected organs on the long-term survival of patients with MVR remains unclear. Historically, the resection of more than one organ has been associated with a poor prognosis and an increased incidence of intraoperative complications [12,22]. Nevertheless, a recently published landmark study by Yang et al. demonstrated that the survival of patients with gastrectomy along with the resection of one organ had no significant difference compared to gastrectomy combined with the resection of more than one organ [21]. In the present systematic review, the mean survival of patients undergoing gastrectomy in combination with a solitary additional organ was 62.2%, 40.9% and 24.9% at 1, 3 and 5 years from the time of surgery, respectively. Unsurprisingly, patients who underwent MVR of >1 organ appeared to have an inferior prognosis (1-year survival: 53.6%, 3-year survival: 24.9% and 5-year survival: 11%). Overall, our data synopsis suggests that the number of resected organs should not be considered a contraindication by definition for gastric cancer surgery [8,17,18].

Tumor boards should be cautious when evaluating patients as potential candidates for multivisceral resection. Importantly, peritoneal dissemination is a classical marker of stage IV disease and of a dismal prognosis [16,27]. In this systematic review, lymph node involvement was found to be a poor prognostic factor in nine studies, increased tumor size in four studies and pancreatic involvement in two studies.

The current systematic review has certain limitations that should be acknowledged. The vast majority of the included studies were retrospective studies from single institutions that were published in different time periods with an evolving AJCC/TNM classification system. Secondly, although our intention was to analyze post-MVR outcomes strictly in the setting of T4b LAGC, our patient cohort included T3 and T4a lesions that were misclassified as T4b based on preoperative imaging. Thirdly, MVR is an unavoidably broad term. For instance, a small transverse colon resection is markedly different than a distal pancreatectomy and splenectomy. Unfortunately, limiting our analysis to major MVR was impossible, since granular subgroup data were not provided within the published studies. Fourthly, significant breakthroughs in surgical, anesthetic and medical management techniques have been introduced throughout the years. The heterogeneity of such robust changes could not be accounted for in the present work but, undoubtedly, should be taken into consideration by practicing surgeons looking for actionable information in our systematic review. More specifically, although neoadjuvant chemotherapy constitutes the initial therapeutic approach of these patients in recent years, few studies evaluated the role of adjuvant or neoadjuvant therapy in patients that underwent MVR resection. Finally, although we initially sought to meta-analyze our data, unfortunately, this could not be performed due to the high degree of heterogeneity in the reporting of most of our outcomes of interest (complications and survival rates).

## 5. Conclusions

Gastrectomy combined with MVR should be considered in patients with T4b LAGC independently from the type and number of resected organs. Surgeons should attempt to recognize true tumor invasions to adjacent organs. Furthermore, the patient selection for MVR should take into account both the patient and tumor characteristics, aiming to recognize true tumor invasions to the adjacent organs, since surgery remains the only potentially curative option for this aggressive cancer. Well-designed studies are needed to further elucidate the role of MVR in patients with T4b LAGC.

## Figures and Tables

**Table 1:** Lesion locations in patients undergoing multivisceral resection (MVR).

Authors	Location n, % (95% CI).			
	Upper	Middle	Lower	Whole
**Aversa et al.** [6]	21, 11%, (7.24–16.29)	46, 24% (18.55–30.6%)	124, 64.9% (57.9–71.3)	NM
**Carboni et al.** [4]	18, 29.51% (19.5–41.95)	31, 50.8% (38.6–62.9%)	12, 19.7% (11.5–31.5)	2, 3.3% (0.25–11.85)
**Cheng et al.** [15]	36, 39.56% (30.12–49.84)	12, 13.2% (7.6–21.8%)	35, 38.5% (29.1–48.7)	8, 8.8% (4.3–16.6)
**Isozaki et al.** [16]	26, 19.85% (13.87–27.55)	45, 34.35% (26.8–42.8%)	45, 34.35% (26.8–42.8)	15, 11.45% (6.96–18.14)
**Jeong et al.** [17]	20, 41.67% (28.8–55.7)	11, 22.9% (13.15–36.7%)	13, 27% (16.46–41.1)	4, 8.33% (2.76–20.1)
**Kim et al.** [18]	5, 14.7% (6–30.6)	8, 23.5% (12.2–40.2%)	13, 38.2% (23.9–55%)	8, 23.5% (12.2–40.2)
**Min et al.** [19]	24, 9.9% (6.7–14.3)	58, 23.9% (18.9–29.6%)	146, 60% (53.8–66%)	15, 6.2% (3.7–10)
**Pacelli et al.** [8]	30, 30.9% (22.6–40.7)	45, 46.4% (36.8–56.3)	22, 22.7% (15.4–32%)	NM
**Molina et al.** [9]	16, 45.7% (30.46–61.8)	13, 37.1% (23.1–53.7%)	6, 17.1% (7.7–33.06%)	NM
**Wang et al.** [20]	NM	12, 30% (18–45.5%)	22, 55% (39.8–69.3%)	6, 15% (6.7–29.5)
**Yang et al.** [21]	48, 36.6% (28.9–45.2)	40, 30.5% (23.3–38.9%)	43 (32.8% (25.4–41.3%)	NM
**Mita et al.** [22]	12, 29.3% (17.5–44.6)	10, 24.4% (13.65–39.5%)	15, 36.6% (23.55–51.9%)	4, 9.8% (3.3–23.1)
**Xiao et al.** [10]	24, 38.1% (27.1–50.5)	17, 27% (17.5–39.1%)	15, 23.8% (14.9–35.7)	7, 11.1% (5.2–21.5)
**Xiao et al.** [23]	18, 28.13% (18.5–40.2)	20, 31.25% (21.2–43.4%)	26, 40.6% (29.45–52.87)	NM
**Ozer et al.** [12]	11, 19.6% (11.2–32)	26, 46.4% (34–59.3)	19, 33.93% (22.9–47.04%)	NM
**Saito et al.** [24]	16, 29.1% (18.7–42.2)	9, 16.36% (8.6–28.5)	21, 38.2% (26.5–51.4)	9, 16.4% (8.6–28.5)
**Sahakyan et al.** [11]	15, 17.05% (10,5–26,35)	28, 31.82% (23–42.16)	30, 34.1% (25–44.5)	14, 50% (39.8–60.2)
**Total:**	340, 23.1% (21.04–25.35%)	431, 29.4% (27.05–31.70%)	607, 41.3% (38.8–43.83%)	92, 6.3% (5.13–7.62%)

**Table 2:** Type and number of organs resected during MVR.

Authors	Organs Resected n, % (95% CI)						
	SP	C	L	P	SB	GB	O
**Amin et al.** [5]	12, 42.86% (26.49–60.95%)	11, 39.29% (23.52–57.63%)	3, 10.71% (2.9–28.01%)	2, 7.14% (0.9–23.73%)	-	-	-
**Carboni et al.** [4]	34, 33.01% (24.66–42.58%)	16, 15.53%, (9.69–23.86%)	12, 11.65% (6.65–19.41%)	28, 27.18% (19.49–36.52%)	1, 0.97% (0.01–5.83%)	2, 1.94% (0.1–7.23%)	10, 9.71% (5.19–17.13%)
**Cheng et al.** [15]	46, 24.08% (18.55–30.64%)	24, 12.57%, (8.53–18.07%)	16, 8.38% (5.14–13.26%)	54, 28.27%, (22.35–35.05%)	12, 6.28% (3.53–10.76%)	13, 6.81% (3.92–11.39%)	6, 3.14% (1.29–6.84%)
**Colen et al.** [25]	13, 37.14% (23.12–53.71%)	5, 14.29% (5.78–29.85%)	2, 5.71% (0.62–19.57%)	12, 34.29% (20.76–50.92%)	3, 8.57% (2.21–23.13%)	-	-
**Dias et al.** [26]	32, 26.89% (19.71–35.52%)	29, 24.37% (17.5–32.85%)	14, 11.76% (7.02–18.9%)	44, 36.97% (28.83–45.94%)	-	-	
**Isozaki et al.** [16]	31, 41.33% (30.87–52.64%)	-	-	31, 41.33% (30.87–52.64%)	-	-	13, 17.33% (10.28–27.57%)
**Jeong et al.** [17]	29, 35.37% (25.87–46.18%)	9, 10.98% (5.67–19.77%)	4, 4.88% (1.54–12.26%)	37, 45.12% (34.81–55.87%)	-	-	5, 6.1% (2.3–13.82%)
**Kim et al.** [18]	13, 33.33% (20.56–49.09%)	15, 38.46% (24.86–54.13%)	-	10, 25.64% (14.41–41.24%)	-	1, 2.56% (0.01–14.36%)	-
**Kobayasbi et al.** [27]	-	35, 36.46% (27.51–46.45%)	10, 10.42% (5.58–18.3%)	36, 37.5% (28.46–47.5%)	-	-	15, 15.63% (9.59–24.31%)
**Martin et al.** [28]	251, 52.4% (47.93–56.84%)	36, 7.52% (5.45–10.25%)	65, 13.57% (10.78–16.94%)	33, 6.89% (4.92–9.54%)	27, 5.64% (3.87–8.11%)	27, 5.64% (3.87–8.11%)	40, 8.35% (6.17–11.19%)
**Min et al.** [19]	-	169, 65.76% (59.76%, -71.29%)	67, 26.07% (21.07–31.77%)	21, 8.17% (5.35–12.22%)	-	-	-
**Mita et al.** [22]	30, 27.03% (19.60–35.99%)	14, 12.61% (7.54–20.18%)	48, 43.24% (43.40–52.54%)	12, 10.81% (6.15–18.09%)	-	2, 1.80% (0.09–6.74%)	7, 6.31% (2.87–12.66%)
**Pacelli et al.** [8]	8, 5.16% (2.48–10.01%)	43, 27.74% (21.28–35.28%)	46, 29.68% (23.03–37.31%)	17, 10.97% (6.87–16.95%)	-	-	41, 26.45% (20.12–33.93%)
**Molina et al.** [9]	12, 23.53% (13.87–36.9%)	6, 11.76% (5.14–23.75%)	17, 33.33% (21.92–47.08%)	10, 19.61% (10.82–32.65%)	-	-	6, 11.76% (5.14–23.75%)
**Shchepotin et al.** [29]	150, 25.13% (21.81–28.76%)	159 (26.63%, 23.24–30.32%)	187 (31.32%, 27.73–35.16%)	101 (16.92%, 14.12–20.14%)	-	-	-
**Wang et al.** [20]	-	22	-	-	-	-	
**Yang et al.** [21]	86, 33.46% (27.97–39.44%)	43, 16.73% (12.64–21.80%)	81, 31.52% (26.14–37.44%)	15, 5.84% (3.50–9.48%)	-	-	32, 12.45% (8.92–17.09%)
**Mita et al.** [30]	54, 48.21% (39.17–57.37%)	10, 8.93% (4.76–15.82%)	33, 29.46% (21.79–38.50%)	4, 3.57% (1.10–9.12%)	1, 0.89% (0.01–5.38%)	1, 0.89% (0.01–5.38%)	9, 8.04% (4.11–14.75%)
**Xiao et al.** [10]	27, 28.13% (20.07–37.86%)	23, 23.96% (16.47–33.45%)	30, 31.25% (22.82–41.12%)	16, 16.67% (10.42–25.48%)	-	-	-
**Ozer et al.** [12]	-	18, 28.13% (18.53–40.20%)	32, 50.00% (38.10–61.90%)	8, 12.50% (6.22–23.03%)	-	1, 1.56% (0.01–13.45%)	5, 7.81% (3.00–17.40%)
**Sahakyan et al.** [11]	34, 26.77% (19.81–35.10%)	23, 18.11% (12.32–25.77%)	30, 23.62% (17.04–31.76%)	16, 12.60% (7.81–19.59%)	8, 6.30% (3.05–12.12%)	-	16, 12.60% (7.81–19.59%)
**Tran et al.** [31]	76, 48.41% (40.72–56.17%)	19 (12.10%, 7.81–18.21%)	42 (26.75%, 20.42–34.19%)	20 (12.74%, 8.33–18.93%)	-	-	-
**Total:**	938, 28.98% (27.44–30.56%)	729, 22.52% (21.11–23.99%)	739, 22.83% (21.42–24.31%)	527, 16.28% (15.05–17.59%)	52, 1.61% (1.22–2.10%)	47, 1.45% (1.09–1.93%)	205, 6.33% (5.54–7.23%)

**Table 3:** Survival of patients after multivisceral resection.

5	Survival after MVR			Survival after 1 Organ Resected			Survival after >1 Organs Resected		
	1-Year	3-Year	5-Year	1-Year	3-Year	5-Year	1-Year	Year	5-Year
**Carboni et al.** [4]	NM	NM	21.8%	NM	NM	NM	NM	NM	NM
**Cheng et al.** [15]	55.2%	22.4%	12.2%	NM	NM	NM	NM	NM	NM
**Dias et al.** [26]	NM	NM	53.4%	NM	NM	NM	NM	NM	NM
**Isozaki et al.** [16]	NM	NM	35%	NM	NM	40%	NM	NM	10%
**Jeong et al.** [17]	74.0%	56.5%	47.5%	NM	NM	NM	NM	NM	NM
**Kim et al.** [18]	NM	NM	37.8%	NM	NM	NM	NM	NM	NM
**Kobayasbi et al.** [27]	59.8%	40.9%	31.1%	NM	NM	NM	NM	NM	NM
**Korenaga et al.** [32]	NM	NM	NM	78.2%	54.2%	39.5%	42.9%	21.4%	21.4%
**Min et al.** [19]	NM	NM	37%	NM	NM	NM	NM	NM	NM
**Mita et al.** [22]	NM	NM	NM	82.5%	47.4%	NM	65.4%	38.1%	NM
**Pacelli et al.** [8]	60.7%	30.3%	27.2%	NM	NM	32.5%	NM	NM	17.2%
**Molina et al.** [9]	88%	51%	34%	NM	NM	NM	NM	NM	NM
**Shchepotin et al.** [29]	NM	NM	25%	NM	NM	NM	NM	NM	NM
**Wang et al.** [20]	75%	49.2%	36.9%	NM	NM	NM	NM	NM	NM
**Yang et al.** [21]	56.1%	26.2%	15.4%	59.3%	26.9%	17.3%	50%	18.1%	6.9%
**Ozer et al.** [12]	53.3%	36%	28.1%	62.3%	40.8%	NM	30.0%	6.4%	NM
**Sahakyan et al.** [11]	NM	18%	10.8%	NM	NM	NM	NM	NM	NM
**Total:**	65.2% (62.61–67.8%)	33.05% (30.71–35.5%)	30.21% (28.25–32.25%)	64.46% (60.39–68.37%),	42.33% (38.19–46.43%),	32.33% (28.95–35.9%)	47.1% (40.58–53.63%)	21% (15.96–26.67%)	15.17% (11.3–20%)

**Table 4:** Poor prognostic factors of the included studies.

Authors	Univariate Analysis	Multivariate Analysis
**Cheng et al.** [15]	N3 status N ratio > 0.3 Bormann type IV R1 resection Lymphatic invasion Perineural invasion Pancreas invasion No liver invasion	Bormann type Curative resection Perineural invasion Nodal status No liver invasion
**Isozaki et al.** [16]	Bormann type 4 Whole stomach Upper-third stomach Dimension of tumor > 90 mm >2 invaded organs N3 status	Location of tumor Histological depth of invasion
**Jeong et al.** [17]	N3 status Lympho-vascular invasion	Lymphatic invasion
**Kobayasbi et al.** [27]	Poor differentiation Extensive vascular invasion Lymph vessel invasion Peritoneal dissemination	Peritoneal dissemination Lymph node ratio > 0.2 Poor differentiation
**Min et al.** [19]	Bormann IV Undifferentiated N3 status Pancreatic invasion	Pancreatic invasion
**Mita et al.** [22]	N3 status R1 resection Organs resected >= 2 Pancreatic resection Spleen resection	R1 resection
**Pacelli et al.** [8]	Peritoneal resection N + status R + resection	N + status Peritoneal resection R + resection
**Molina et al.** [9]	Lymph nodes involvement	Lymphatic invasion R1 resection
**Wang et al.** [20]	Tumor size (>9 cm) Advanced T stage (pT4b) Lymph node metastasis	Advanced T stage (pT4b) Lymph node metastasis
**Yang et al.** [21]	Pancreas resection Spleen resection Resection of >15 lymph nodes Vascular tumor emboli R+ resection	R+ resection Vascular tumor emboli Lymph nodes > 15
**Xiao et al.** [10]	Total gastrectomy Whole gastric location R1 resection	R1 resection Linitis plastica
**Xiao et al.** [23]	Tumor > 7 cm R+ resection	Tumor > 7 cm Non-curative resection
**Ozer et al.** [12]	Age > 70 y >2 organs resected Positive lymph node metastasis Presence of comorbidities	Age older > 70 y Lymphatic invasion Number of organs resected >2
**Sahakyan et al.** [11]	Total gastrectomy Obesity (BMI < 30) N3 status	Obesity (BMI < 30) Nodal stage (N3)

## Data Availability

The data that support the findings of this study are openly available in Zenodo at https://doi.org/10.5281/zenodo.8275401, accessed on 23 August 2023.

## Supplementary Materials

The following supporting information can be downloaded at https://www.mdpi.com/article/10.3390/jcm12237360/s1: Supplementary Table S1: Quality assessment of the included case series using the NHLBI Quality Assessment Scale for case series. Supplementary Table S2: Survival rates based on R resection.
