# Supplementary material for: Multivisceral Resection for Locally Advanced Gastric Cancer: A Systematic Review and Evidence Quality Assessment
Source: J Clin Med. 2023 Nov 28;12(23):7360. doi: 10.3390/jcm12237360 (PMC10707710; doi:10.3390/jcm12237360)
Supplement: Supplementary file 1 [file jcm-12-07360-s001.zip › jcm-2667960-supplementary.pdf]

Supplemental Table 1. Quality assessment of included case series using the NHLBIQuality Assessment Scale for case series.

First Author	Was the study objective clearly stated?	Was the study population clearly and fully described, including a case definition?	Were the cases consecutive?	Were the subjects comparable?	Was the intervention clearly described?	Were the outcome measures clearly defined, valid, reliable, and implemented consistently across all study participants?	Was the length of follow-up adequate?	Was the methodology well-described?	Were the results well-described?	Total score	Quality rating
**Amin**	1	0	1	1	1	1	1	1	0	7	Good
**Aversa**	1	1	1	1	0	1	0	1	1	7	Good
**Carboni**	1	1	1	1	1	1	1	1	1	9	Good
**Cheng**	1	1	1	1	1	1	1	1	1	9	Good
**Colen**	1	1	1	1	1	1	0	1	1	8	Good
**Dias**	1	1	1	1	1	1	1	1	1	9	Good
**Isozaki**	1	1	1	1	1	1	1	1	1	9	Good
**Jatzko**	1	0	1	1	0	1	0	1	0	5	Fair
**Jeong**	1	1	1	1	1	1	1	1	1	9	Good
**Kim**	1	1	1	1	1	1	1	1	1	9	Good
**Kobayasbi**	1	1	1	1	1	1	1	1	1	9	Good
**Korenaga**	1	1	1	1	0	1	1	1	1	8	Good
**Martin**	1	1	1	1	0	1	1	1	0	7	Good
**Min**	1	1	1	1	1	1	1	1	1	9	Good
**Mita**	1	1	1	1	1	1	1	1	1	9	Good
**Pacelli**	1	1	1	1	1	1	1	1	1	9	Good
**Molina**	1	1	1	1	1	1	1	1	1	9	Good
**Shchepotin**	1	1	1	1	1	1	1	1	0	8	Good
**Wang**	1	1	1	1	1	1	1	1	0	8	Good
**Wang**	1	1	1	1	1	1	1	1	1	9	Good
**Zhang**	1	1	1	1	1	1	0	1	0	7	Good
**Yang**	1	0	1	1	1	1	1	1	1	8	Good
**Mita**	1	1	1	1	1	1	1	1	1	9	Good

**L. Xiao**	1	1	1	1	1	1	1	1	1	9	Good
**H. Xiao**	1	1	1	1	1	1	1	1	1	9	Good
**Ozer**	1	1	1	1	1	1	1	1	1	9	Good
**Saito**	1	1	1	1	1	1	0	1	1	8	Good
**Sahakyan**	1	1	1	1	1	1	1	1	1	9	Good
**Ocana**	1	0	1	1	0	1	1	1	0	6	Fair
**Tran**	1	1	1	1	1	1	1	1	1	9	Good

The NHLBI scale ranges from 1-9; with a score of 1-3 denoting poor quality, 4-6 fairquality,and 7-9 suggesting high quality.
NHLBI: National Heart, Lung, and Blood Institute

Supplemental Table 2: Survival rates based on R resection.

Authors	Survival based on R resection	
	**R0 resection**	**R+ resection**
**Carboni et al. (4)**	R0= 30.6% (p=0.001)	R+=0%
**Dias et al. (26)**	R0=56.9%	R+=28.6%
**Kobayasbi et al. (27)**	R0 better survival (P=0.004)	NM
**Mita et al. (22)**	R0=78.3% (1-year)<br>R0= 47.7% (3-year) (P<0.002)	R1= 46.6% (1-year)<br>R1= 14.3% (3-year)
**Pacelli et al. (8)**	R0= 43.7% (5-year) (P=0 .001)	R1=31.4 % (5-year)
**Wang et al. (20)**	R0-resection (P=0.0174)	NM
**Yang et al. (21)**	R0 better survival (P < 0.05)	NM
**Mita et al. (30)**	R0=78.1 (1-year) (P .001)<br>R0= 62.1 (3-year) (P .001)	R+=8.6% (1-year)<br>R+=0 (3-year)
**Xiao et al.(10)**	R0=68.2% (1-Year) (P =0.030)	R+=41.9% (1-year)
**Xiao et al. (23)**	R0=34 months (P = 0.004)	R1=11 months
